# Supplementary figures and images for: Autophagy inhibition overcomes multiple mechanisms of resistance to BRAF inhibition in brain tumors
Source: eLife. 2017 Jan 17;6:e19671. doi: 10.7554/eLife.19671 (PMC5241115; doi:10.7554/eLife.19671)

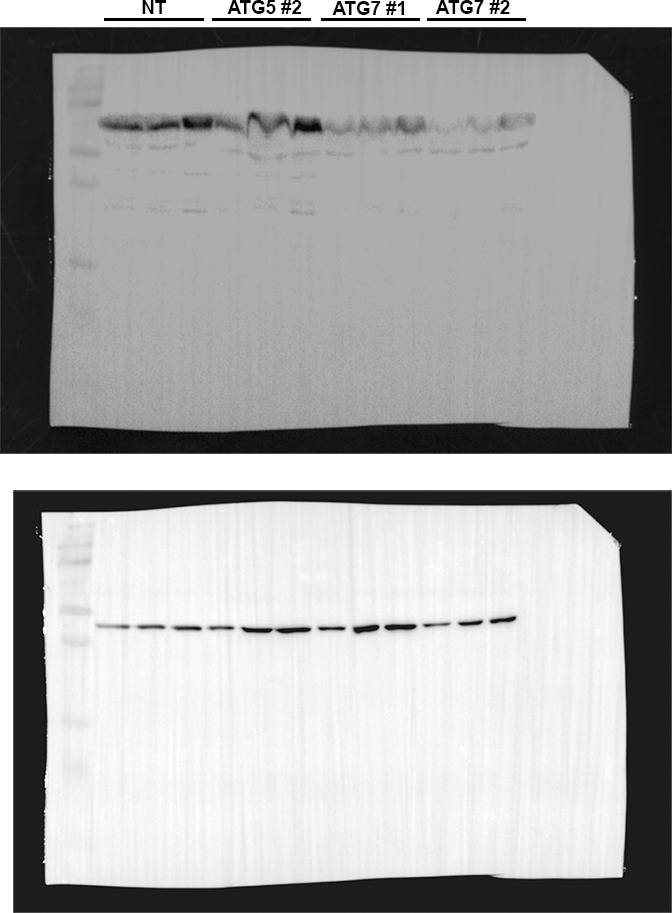

Supplement: Figure 8—figure supplement 1—source data 1. — All ATG7 bands shown were run and developed on the same blot. DOI: http://dx.doi.org/10.7554/eLife.19671.024 [file elife-19671-fig8-figsupp1-data1.jpg]
